# Investigation of Multi-Frequency SAR Data to Retrieve the Soil Moisture within a Drip Irrigation Context Using Modified Water Cloud Model

**DOI:** 10.3390/s22020580

**Published:** 2022-01-12

**Authors:** Emna Ayari, Zeineb Kassouk, Zohra Lili-Chabaane, Nicolas Baghdadi, Mehrez Zribi

**Affiliations:** 1 CESBIO (CNRS/UPS/IRD/CNES/INRAE), 18 Av. Edouard Belin, bpi 2801, CEDEX 9, 31401 Toulouse, France; ayari.emna.inat@gmail.com; 2 National Agronomic Institute of Tunisia, Carthage University, LR17AGR01 InteGRatEd Management of Natural Resources: remoTE Sensing, Spatial Analysis and Modeling (GREEN-TEAM), Tunis 1082, Tunisia; zeineb.kassouk@inat.u-carthage.tn (Z.K.); Zohra.LiliChabaane@inat.u-carthage.tn (Z.L.-C.); 3 CIRAD, CNRS, INRAE, TETIS, University of Montpellier, AgroParisTech, CEDEX 5, 34093 Montpellier, France; nicolas.baghdadi@teledetection.fr

**Keywords:** ALOS-2, Sentinel-1, soil moisture, row vegetation, modified water cloud model, drip irrigation

## Abstract

The objective of this paper was to estimate soil moisture in pepper crops with drip irrigation in a semi-arid area in the center of Tunisia using synthetic aperture radar (SAR) data. Within this context, the sensitivity of L-band (ALOS-2) in horizontal-horizontal (HH) and horizontal-vertical (HV) polarizations and C-band (Sentinel-1) data in vertical-vertical (VV) and vertical-horizontal (VH) polarizations is examined as a function of soil moisture and vegetation properties using statistical correlations. SAR signals scattered by pepper-covered fields are simulated with a modified version of the water cloud model using L-HH and C-VV data. In spatially heterogeneous soil moisture cases, the total backscattering is the sum of the bare soil contribution weighted by the proportion of bare soil (one-cover fraction) and the vegetation fraction cover contribution. The vegetation fraction contribution is calculated as the volume scattering contribution of the vegetation and underlying soil components attenuated by the vegetation cover. The underlying soil is divided into irrigated and non-irrigated parts owing to the presence of drip irrigation, thus generating different levels of moisture underneath vegetation. Based on signal sensitivity results, the potential of L-HH data to retrieve soil moisture is demonstrated. L-HV data exhibit a higher potential to retrieve vegetation properties regarding a lower potential for soil moisture estimation. After calibration and validation of the proposed model, various simulations are performed to assess the model behavior patterns under different conditions of soil moisture and pepper biophysical properties. The results highlight the potential of the proposed model to simulate a radar signal over heterogeneous soil moisture fields using L-HH and C-VV data.

## 1. Introduction

Over the last few decades, the agricultural sector water demand has increased to ensure food security for a growing population [1,2]. In arid and semi-arid areas, the irrigated sector has increased the pressure on water resources under climatic irregularities, i.e., successive years of drought or flooding [1,3,4]. This critical situation becomes increasingly complicated with the insufficient use of water in agricultural fields [5,6]. To improve water resource management in the agricultural sector, soil moisture estimation is a key component to optimize irrigation scheduling and precision irrigation [7,8].

Remote sensing has demonstrated a high potential to retrieve soil moisture from agricultural field scales to regional scales owing to the resolution and repetition frequency of remote-sensing data acquisitions compared to punctual measurements of the soil moisture [9,10,11,12]. Based on synthetic aperture radar (SAR) data, many studies have been devoted to soil moisture estimation in the X-, C- and L-bands [12,13,14,15,16,17,18,19,20,21,22,23,24,25,26,27,28,29]. The developed approaches to estimate soil moisture using SAR data generally have not considered the heterogeneity of soil moisture at a pixel scale which remains a persistent challenge.

To relate radar signals to in situ measurements, such as soil moisture and roughness [30] and biophysical vegetation parameters across covered fields, different radar backscattering models have been employed [31,32]. Some of the aforementioned models are based on physical approaches, such as the Karam model based on the physical interactions of electromagnetic waves with vegetation [33,34], and semi-empirical approaches, such as the Michigan microwave canopy scattering model that divides the vegetation canopy into multilayer compositions [31,35,36,37,38] and the water cloud model (WCM) [18,27,39,40,41,42,43,44,45,46]. 

The simplicity of the WCM makes it widely used in the literature where backscattering is the sum of the contributions of three components, namely, vegetation, soil scattering attenuated with the vegetation effect and soil-vegetation interactions. Bare soil backscattering is calculated through a wide range of models coupled to the WCM including physical approaches, such as the geometric optic model (GOM) [21], integral equation model (IEM) [47], AIEM [28,48], and IEM modified by Baghdadi (IEM-B) [18,49], and semi-empirical approaches, such as the Oh model [50,51,52] or empirical models requiring calibration and validation (particularly exponential [21,53] or linear relationships [43,44], respectively).

In previous studies relying on the WCM, the soil–vegetation interaction term has generally not been considered. However, some studies have proposed different expressions for this term, e.g., as a function of the Fresnel reflectivity and volume scattering coefficients in [36,37] or via calibrated semi-empirical equations in [21,49]. To evaluate the vegetation effect, various studies have explored the impact of canopy descriptors such as biophysical parameters (crop height, leaf area index, biomass, vegetation coverage fraction or vegetation water content) [38,51,54], SAR polarization ratios [51,55,56,57,58] or indices derived from optical images, such as the normalized difference vegetation index (*NDVI*) [18,43,44,49,59].

To estimate the soil moisture within a homogenous vegetation context, several techniques have been employed to invert models, including direct inversion [44,49], change detection [29,60,61], Bayesian approaches [62], look-up tables [49,52] and artificial neural networks (ANNs) [23,63,64,65,66,67].

WCM parametrization of vegetation and soil variables has been performed based on experimental data relating the model performance to the quality and robustness of in situ data [32]. The scientific community has dedicated considerable effort to improving the accuracy of the WCM. Therefore, different versions of the WCM have been reported in the literature, demonstrating a notable improvement in the simplified WCM under corresponding assumptions [16,38,47,68,69]. Zhang et al. [21] suggested a modified version of the WCM using the vegetation proportion based on the hypothesis that vegetation does not cover pixels with the same proportion across the various growth stages. As a result, the total backscattering signal was determined as the sum of the vegetation coverage fraction contribution and the proportion of bare soil backscattering. The obtained soil moisture retrieval results using artificial neural networks (ANNs) trained by X-band data yielded maximum RMSE values of 4.3 vol.%, 4.6 vol.%, and 6.4 vol.% for the three corn growth stages: emergence, trefoil, and jointing stages, respectively. 

He et al. [47] proposed a modified WCM under relatively sparse vegetation conditions. They separated the contributions of covered and underlying ground according to the vegetation coverage fraction calculated as a function of the normalized difference vegetation index. Consequently, using C-band SAR data, the backscattering coefficients modeled with a modified version of the WCM achieved a higher sensitivity to patchy vegetation conditions than did the simplified WCM. The RMSE value, characterizing the relationships between the measured backscattering and predicated coefficients, decreased from 2.04 dB to 1.40 dB and from 2.45 dB to 1.69 dB in HH and VV polarizations, respectively. This approach provided a higher soil moisture estimation accuracy, and the RMSE values were smaller than 3.4 vol.%. Bao et al. [16] proposed an improved WCM based on the Taylor approximation using Sentinel-1 data. Model validation yielded an RMSE value of approximately 5.3 vol.% for soil moisture estimation.

Most studies have been developed to retrieve soil moisture using C- and X-band SAR data rather than L-band data due to the limited availability of SAR data at the L-band frequency. The Japan Aerospace Exploration Agency (JAXA) provides Advanced Land Observing Satellite (ALOS) and ALOS-2 L-band images on request. In the near future, the European Space Agency (ESA) will deploy the Radar Observing System for Europe–L-band (ROSE-L) Mission for land and geohazard monitoring, where soil moisture retrieval is one of the main objectives [70]. Therefore, actual scientific research should consider the potential of L-band data to estimate soil parameters, such as the roughness and soil moisture content.

Regarding covered fields, Zribi et al. [18] explored the potential of ALOS-2 L-band data to estimate the soil moisture in tropical areas, as applied to turmeric and marigold vegetation, thereby providing an accuracy lower than 8.7 vol.% and 11 vol.% in HH and HV polarizations, respectively. In semi-arid areas, Ayari et al. [49] proposed an approach to retrieve the soil moisture content in cereal fields using the NDV as a vegetation descriptor in the WCM with L-band data rather than C-band data. The developed approach improved the soil moisture estimation accuracy using L-band data in HV polarization, and the root mean square error (RMSE) value was smaller than 7 vol.%, thus highlighting the potential of the IEM-B model coupled with the WCM for soil moisture estimation in the HH polarization case. Wang et al. [52] focused on WCM parametrization using L-band data as a vegetation descriptor under VH polarization. The overall accuracy of soil moisture estimation was improved, and the unbiased root mean squared error (ubRMSE) values on soil moisture were smaller than 9.8 vol.% across different crop types (canola, corn, beans, and wheat).

Referring to the previously mentioned works, the L-band data potential for soil moisture estimation should be explored within other contexts and assumptions. For example, radar signal modeling across spatially heterogeneous agricultural fields remains a challenge, as most approaches have been developed for homogeneous crop fields, such as cereal fields. Furthermore, localized irrigation is a source of complexity in SAR signal modeling and subsequent soil moisture estimation. In the case of drip irrigation, soil moisture is not distributed homogeneously across the entire plot [71]. 

Hence, this work aims to compare the L-band and the C-band potentials for soil moisture retrieval under the same conditions, i.e., locally irrigated heterogeneous crops using a modified version of the water cloud model. This article is divided into five sections. Section 2 describes the study zone and the dataset used, including satellite images, and gathered data (soil properties and biophysical parameters) and presents the proposed methodology for radar signal modeling and soil moisture estimation. Section 3 details the results and discusses them and is divided into four parts: radar sensitivity to soil moisture heterogeneity, radar sensitivity to heterogeneous vegetation cover parameters, calibration, and validation of the modified WCM and sensitivity of the modified WCM to soil moisture. Section 4 draws conclusions.

## 2. Materials and Methods

### 2.1. Study Site

**Figure 1:**
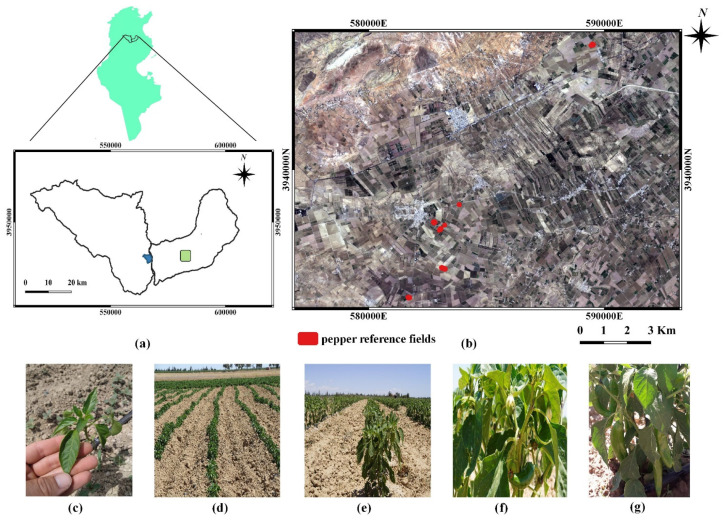
(**a**) Location of the study area in the Kairouan Plain at the center of Tunisia (red mark). (**b**) Reference pepper fields in the study area delimited by Sentinel-2 images in yellow. Photos of selected pepper growth stages in the reference fields (**c**) leaf development stage (**d**) pepper crop rows during leaf development stage (**e**) flowering stage (**f**) fruit development stage (**g**) fruit ripening stage.

The present study was carried out in a semi-arid area: the Merguellil Plain (9°230–10°170 E, 35°10–35°550 N) in the Kairouan Plain, central Tunisia, as shown in Figure 1. The Kairouan Plain is a flat landscape extending over 3000 km² receiving approximately 300 mm of precipitation annually with a rainy season ranging from September to April and a summer season with almost no rainfall. The temperature varies between 11 and 30 °C. The Kairouan Plain water table is fed by surface infiltration and transferred from several water tables, such as Bouhaffna, Haffouz-Chrichira and Ain El Bidha. The study area is characterized by large, irrigated fields of cereal, olive tree and market garden crops [72]. As a result, the irrigation water demand increase has enhanced the pressure exerted on the aquifer, leading to overexploitation reflected by a decrease in the water level between 0.25 and 1 m per year [73].

In the study area, pepper is a leading seasonal garden crop in the summer. For three months starting from June, reference fields soil is ploughed and fertilized to prepare seed beds. After transplanting, pepper seedlings were drip irrigated. The drip irrigation is supplied by lines of irrigation tape and spaced emitters. Subsequently, leaves start to develop until inflorescence emergence and flowering. Afterwards, the flowers transform to fruits. The mature peppers are harvested multiple times until the senescence of pepper plants. 

### 2.2. Dataset Description

#### 2.2.1. Satellite Images

(a) Optical data: Sentinel-2

After the launch of Sentinel-2 (S-2) A and B on 23 June 2015 and 7 March 2017, respectively, optical data became free and open access with a spatial resolution varying between 10 m ×10 m and 60 m × 60 m, and a revisit time of up to 5 days in 13 spectral bands at visible and mid-infrared wavelengths. In the present study, we used S-2 surface reflectance products downloaded from the Theia site (https://www.theia-land.fr/, accessed on 8 January 2022), already orthorectified and atmospherically corrected with a mask of clouds and shadows owing to the MAJA algorithm. 

On each acquisition date and using red visible and near infrared bands with center wavelengths of approximately 665 and 833 nm, respectively, we calculated the NDVI and averaged this index for each reference field as expressed in the following equation [18,44,74,75]:

(1) $$NDVI=\frac{R_{NIR}-R_{Red\;}}{R_{NIR}+R_{Red\;}}$$

where $R_{NIR}$ and $R_{Red\;}$ are the surface reflectance in the two bands, near infrared and red visible, respectively [76]. 

**Figure 2:**
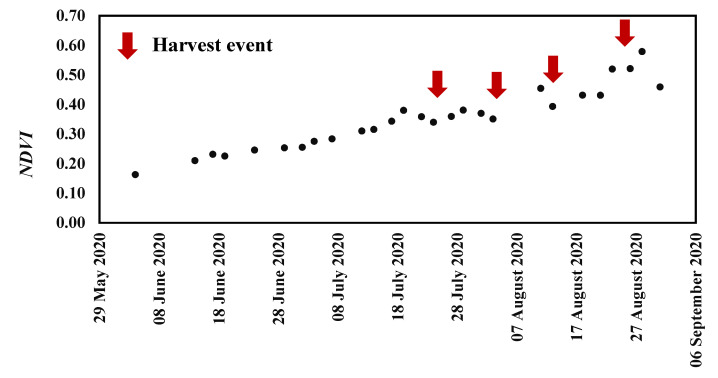
Normalized difference vegetation index (*NDVI*) values evolution during the summer season of 2020 over a pepper reference filed in the Kairouan Plain where red arrows indicate pepper harvest accompanied by hand weeding.

Figure 2 describes the evolution of NDVI values during the summer season over one pepper field. NDVI values are varying between 0.16 and 0.38 corresponding to the development of pepper plant leaves and fruit ripening, respectively, starting from June until the middle of July. NDVI values decrease to 0.34 corresponding to the first fruit harvest date. Afterwards, NDVI values oscillate between 0.34 and 0.58 where minimum values mark harvest events as identified by red arrows in the figure accompanied with manual weedings.

(b) SAR data

- Sentinel-1

Eight C-band SAR images were acquired by Sentinel-1 A (S-1 A) and B (S-1 B) constellation between June and August 2020 over the study area, as described in Table 1. Sentinel-1 data are produced as ground range-detected (GRD) data and generated in the interferometric wide-swath (IW) mode. Owing to the launch of Sentinel-1 A and B sensors, C-band data are available in vertical-vertical (VV) and vertical-horizontal (VH) polarizations at an incidence angle of approximately 39° for the Kairouan plain site, with a spatial resolution of 10 m × 10 m and a revisit time of up to 6 days. In regard to the S-1 products, several processing steps were carried out involving thermal noise removal, radiometric calibration, terrain correction based on a Shuttle Radar Topography Mission (SRTM) digital elevation model (DEM) with a 30 m resolution and speckle filtering with a Lee filter.

**Figure 3:**
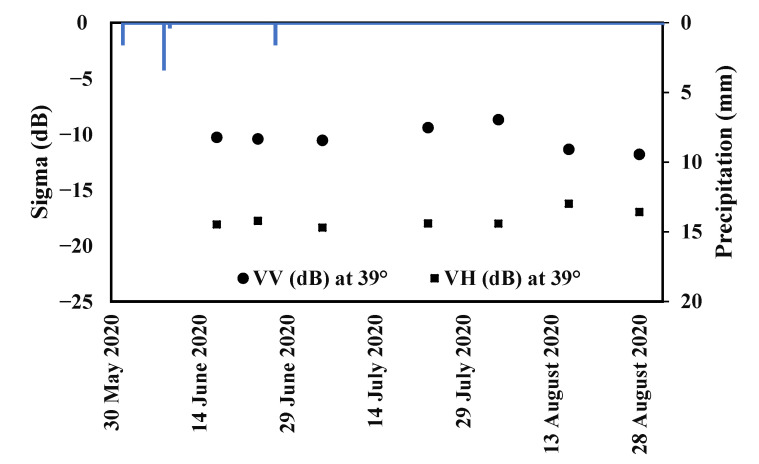
C-band Sentinel-1 backscattering coefficients (sigma) in dual-polarization VV and VH at an incidence angle of 39° and the precipitation quantity temporal evolutions during June, July, and August 2020.

Figure 3 shows the temporal variation of Sentinel-1 data for one pepper field during acquisition dates as a function of precipitations recorded by the climatic station of Sidi Ali Ben Salem. According to Figure 3, one rainfall event of approximately 1.7 mm was recorded on 27 June 2020 along the acquisition chronology. Between 5 July 2020 and 4 August 2020, slight variations characterize the temporal evolution of C-VV and C-VH data. Starting from 4 August 2020 to 28 August 2020, C-VV decreased by 3.11 dB when rainfall was absent. From 4 August 2020 to 16 August 2020, C-VH evolution is rather stable, with small oscillations.

- ALOS-2

Advanced Land Observing Satellite-2 (ALOS-2) images are acquired at the L-band frequency by the Phased Array Synthetic Aperture Radar (PALSAR). According to Table 1, five ALOS-2 SAR images were obtained in the stripmap mode between June and August 2020 over the study area. Radar images were available in dual polarizations, i.e., horizontal-horizontal (HH) and horizontal-vertical (HV), at an incidence angle of 32.5° for the study zone with a spatial resolution of 6 m × 6 m and a revisit cycle of 14 days. Radiometric calibration was performed based on the available data to transform digital values into backscattering coefficients on a linear scale. Georeferencing of ALOS-2 data was carried out through a control points method, as detected using *NDVI* images calculated from Sentinel-2 optical data with a root mean square (RMS) control point error smaller than 0.5 pixels [18,49]. 

**Figure 4:**
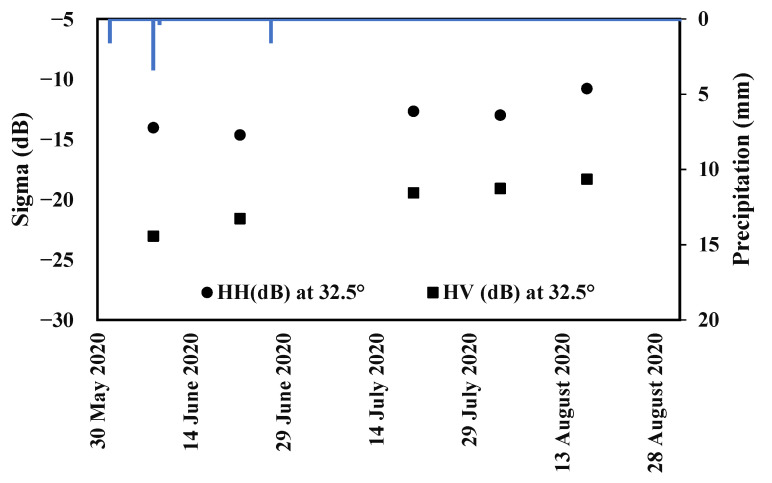
L-band ALOS-2 backscattering coefficients (sigma) in dual-polarization HH and HV at an incidence angle of 32.5° and precipitation quantity temporal evolutions during June, July, and August 2020.

Figure 4 shows the temporal behavior of the ALOS-2 signal as function of recorded precipitations. Three rainfall events marked the chronology on 8 June 2020, 9 June 2020 and 27 June 2020 with by low intensities of 3.41 mm, 0.4 mm, and 1.6 mm, respectively. Between 8 June 2020 and 22 June 2020, the L-HH signal decreased by 0.61 dB and L-HV increased by 1.46 dB. L-band sigma increased by 1.96 and 2.13 dB for L-HH and L-HV, respectively, from 22 June 2020 to 20 July 2020. During precipitation absence between 20 July 2020 and 17 August 2020, ALOS-2 signal increased by 1.90 dB in HH polarization and by 1.14 dB in HV polarization. Due to the limited number of ALOS-2 acquisitions, the interpretation of L-band signal temporal behavior remains complicated in the present case. According to Figure 3 and Figure 4, we can notice the absence of correlations between SAR data evolutions and rainfalls because signals are lower in accordance with precipitations. In this case, the presence of irrigations probably affects the SAR signal more than rainfall.

#### 2.2.2. In Situ Measurements

**Figure 5:**
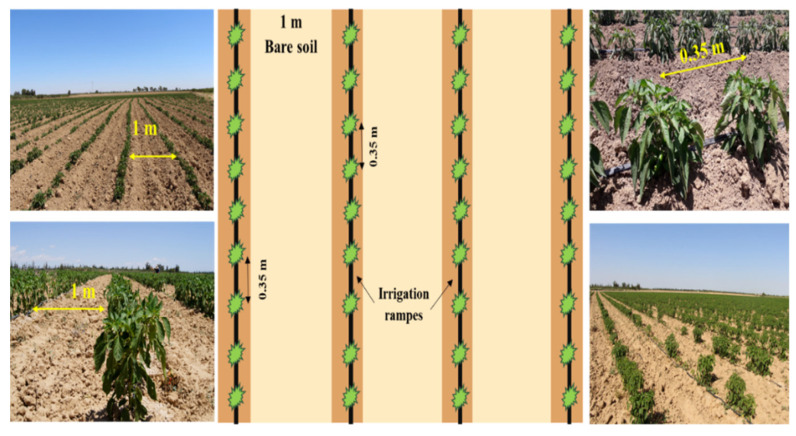
Pepper reference field architecture: delineation of the bare soil and vegetation parts, where the green spots represent pepper plants and the black lines represent the irrigation tape, based on ground truth scaling and illustrations.

During S-1 and ALOS-2 acquisitions (between June and August 2020), data gathering campaigns were conducted in seven irrigated reference pepper fields in the Sidi Ali Ben Salem area in the Kairouan Plain. Reference fields were considered to measure soil surface properties (roughness and soil moisture) and vegetation parameters (leaf area index, fraction cover and height of vegetation cover). The surface area of these fields varied between 1 and 2.6 ha. As shown in Figure 5, the pepper coverage is heterogeneous with a directional vegetation structure. Each pepper field comprised vegetation rows with local drip irrigation. An approximate distance of 0.35 m separated two pepper plants within one row. The inter-row spacing was 1 m and consisted of bare soil.

(a) Soil moisture (Mv)

In the row crop case with drip irrigation, the soil moisture spatial pattern is heterogeneous: the water content in the soil below vegetation is different from that in the bare soil part. As a result, soil moisture measurements were performed at a depth of 5 cm and with a hand-held Theta probe in each test field at 10 points randomly chosen in the bare soil part and 10 points in the soil part below vegetation. The moisture measurements were completed within two hours separating the measurements and the satellite overpass time.

Within this context, we obtained soil moisture measurements collected over bare soils, namely, inter-row soil moisture $Mv_{inter\text{-}row}$ varying between 4.9 vol.% and 31.4 vol.%, and soil moisture measurements collected over the underlying pepper plants soil, namely $Mv_{veg\text{-}row}$ fluctuating between 5.6 vol.% and 32.1 vol.%, during all campaigns, as detailed by the minimum and the maximum values in Table 2. The inter-rows moisture is impacted exceptionally by water loss from irrigation systems which increase the soil moisture values. 

For each reference field, we calculated an average soil moisture value based on the hypothesis that bare soil, characterized by $Mv_{inter\text{-}row}$, covered the proportion α  of the field area and 1−α was covered by rows of pepper plants equipped by irrigation tapes, characterized by $Mv_{veg\text{-}row}$. Consequently, the average soil moisture value at the field scale was calculated with the following expression:

(2) $$\mathrm{Mv}=\;\alpha \times Mv_{inter\text{-}row}+\left(1-\alpha \right)\times Mv_{veg\text{-}row}$$

According to the ground truth observations, we estimated that 15% of the field area is covered by irrigation tapes and emitters. The rest of the area, which is equal to 85%, receives irrigation water through water transfers in the soil. As a result, the measured soil moisture is the result of the contribution of soil moisture next to the emitters and 85% of the soil moisture off the irrigation zone. Therefore, in the present study, we consider that α is equal to 0.85.

(b) Soil roughness

The soil roughness was characterized with a 1 m pin profiler exhibiting a pin spacing of 2 cm using six roughness profiles (three perpendicular and three parallel to the inter-row tillage direction). These profiles were digitized to calculate statistical parameters of the soil roughness, namely, the root mean square of the height (Hrms) and correlation length (Lc), as mentioned in [44,49]. Hrms varied between 1.5 and 2.54 cm, and the Lc value varied between 2.98 and 7.40 cm.

(c) Vegetation height (H)

During SAR data acquisition, as indicated in Table 2, 20 measurements of the pepper height (H) were obtained in each reference field on each date. The vegetation height could reach a maximum value of 0.64 m.

(d) Vegetation cover fraction (*Fc*) and Leaf area index (LAI)

In various studies, the vegetation cover fraction has been calculated as a function of the *NDVI* derived from optical images with the dimidiate pixel model (DPM) [77]. In the present work, we estimated the LAI and vegetation cover fraction for each pepper field through 20 hemispherical digital images, as mentioned in [44,49,74].

**Figure 6:**
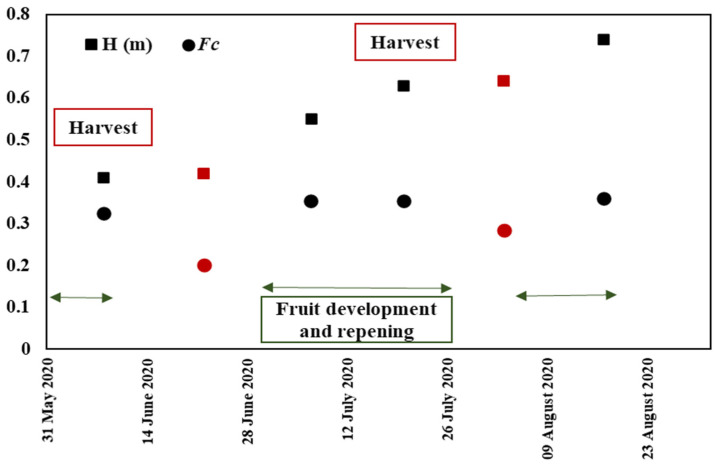
The temporal evolution of pepper biophysical parameters: Vegetation cover fraction (*Fc*) and vegetation height (H) during the pepper growth cycle where red arrows indicate fruit harvest events.

The LAI value varied from 0.07 to 1.75. The pepper cover fraction (*Fc*) value fluctuated across the various growth stages between 0.1 and 0.5, covering the four pepper phenological stages starting with vegetative growth at the end of June, followed by flowering, fruit setting and fruit formation. Figure 6 represents the evolution of *Fc* and H measured over a pepper reference field where harvest event dates were different to the chosen fields in Section 2.2.1 (a). According to Figure 6, the highest values were observed at the flowering and fruit setting stages when the pepper plants reached the maximum leaf growth level. The lowest values characterized fruit harvest events accompanied by hand weeding.

### 2.3. Methodology

In the present section, we detail the adopted methodology to simulate the L-HH and C-VV SAR signal over covered vegetation fields. The reference fields are characterized by spatial heterogenous vegetation coverage which lead to inter-pixel heterogenous spatial repartition of the soil moisture. Therefore, we used a modified version of WCM to consider the different component of pepper fields. The modified WCM is coupled with IEM-B to simulate SAR signal over bare soil. 

#### 2.3.1. Modified Water Cloud Model (WCM)

Attema and Ulaby in 1978 [39] proposed a semi-empirical model, the WCM, as a first-order approximation of the radiation transferred from vegetation canopies by considering that the vegetation response to radar signals consists of effects attributed to soil and the vegetation cover [31,78]. In regard to radar signals (in pq polarization), the total backscattering $\left(\sigma^{0\;}{}_{\mathrm{total},\;\mathrm{pq}}\right)\;$ comprises the vegetation contribution $\left(\sigma_{veg,\;pq}^{0}\right)$, soil contribution attenuated by vegetation $\left(\tau^{2}\sigma_{soil,\;pq}^{0}\right)$ and soil-vegetation term $\left(\;\sigma_{soil\text{-}veg,pq}^{0}\right)$, which has been mostly neglected. As a result, the WCM is expressed with the following equations (Equations (3)–(5)):

(3) $$\sigma^{0\;}{}_{\mathrm{total},\;\mathrm{pq}}=\sigma_{veg,pq\;}^{0}+\tau^{2}\sigma_{soil,pq}^{0}$$

(4) $$\tau^{2}=\exp(-2\times B\times V1\times \sec\theta )$$

(5) $$\sigma_{veg,pq}^{0}=A\times V2\times \cos\theta \times \left(1-\tau^{2}\right)$$

where *A* and *B* are parameters that depend on the characteristics of the vegetation canopy and SAR configurations and *V*1 and *V*2 are vegetation descriptors.

As mentioned above, parameters *V*1 and *V*2 have been determined based on vegetation parameters. Several works have particularly considered information retrieved from optical data to characterize these parameters, especially the *NDVI* [43,44,49].

The *NDVI* values were averaged for each pepper reference field characterized by vegetation rows separated by 1 m of bare soil. Consequently, the calculated *NDVI* values were affected by bare soil describing the total field and not only the vegetation such in homogeneous vegetation cover cases. Additionally, LAI measurements expressed the surface leaf of pepper seedlings per unit of soil area. In a spatial heterogeneous case, the soil area contribution is present and dominates the vegetation presence in the hemispherical photos. In this context, for the present study case, we propose to use the vegetation height as the parameter to describe the cover dynamics outside the bare soil zone. Thus, we consider *V*1 = *V*2 = H in this study as pepper descriptor without the bare soil part. In fields with vegetation crop rows, the above simplified version of the WCM, initially proposed for homogenous regions, is not applicable due to the heterogeneous spatial distribution of pepper plants. Hence, [21,47] introduced ancillary information on the vegetation fraction (*Fc*). Consequently, the vegetation contribution in the WCM was delineated by the cover fraction.

In the study case involving the pepper field organization, we considered a combination of two contributions. The first contribution $\;\sigma_{veg\text{-}row,\;pq\;}^{0}$ is that of the pepper row weighted by the cover fraction (*Fc*), and the other contribution $\sigma_{inter\text{-}row,pq}^{0}$ is that of inter-rows of the pepper part, weighted by (1 − *Fc*):

(6) $$\sigma^{0\;}{}_{\mathrm{field},\mathrm{pq}}=Fc\times \sigma_{veg\text{-}row,pq\;}^{0}+\left(1-Fc\right)\times \sigma_{inter\text{-}row,pq}^{0}$$

where $\sigma_{inter\text{-}row,pq}^{0}$ is the bare soil backscattering term separating two pepper rows simulated with IEM-B and $\sigma_{veg\text{-}row,\;pq\;}^{0}$ is the total contribution of the vegetation rows.

The total contribution of pepper rows $\sigma_{veg\text{-}row,\;pq\;}^{0}$ is the sum of the first term $\sigma_{veg,qp}^{0}\;$ representing the volume scattering contribution of the vegetation and the soil effect attenuated by the vegetation cover $\sigma_{bare\text{-}soil,pq}^{0}$. The bare soil scattering term is divided into two terms: the first term $\;\sigma_{under\text{-}veg\;}^{0}$ corresponds to the irrigated area (under peppers seedlings locally irrigated), and the second term $\;\sigma_{inter\text{-}veg}^{0}\;$ corresponds to the non-irrigated area. This last one is around pepper plants and separating two successive peppers seedlings, relatively far from water emitters and consequently non-irrigated. These two contributions $\;\sigma_{under\text{-}veg\;}^{0}\;$ and $\;\sigma_{inter\text{-}veg}^{0}\;$ are weighted according to the corresponding fractions. As specified above, the locally irrigated area is approximately covering 0.15 m^2^ per one m^2^ ($\frac{0.15}{Fc\;}$) of the row crop area. Therefore, the pepper row vegetation contribution is expressed as:

(7) $$\sigma_{veg\text{-}row,qp}^{0}=\sigma_{veg,qp}^{0}+\tau^{2}\sigma_{bare\text{-}soil,pq}^{0}$$

(8) $$\sigma_{veg\text{-}row,qp}^{0}=\sigma_{veg,qp}^{0}+\tau^{2}(\frac{0.15}{Fc\;}\times \sigma_{under\text{-}veg}^{0}+\frac{Fc-0.15}{Fc}\times \sigma_{inter\text{-}veg}^{0})$$

where $\;\sigma_{under\text{-}veg}^{0}\;$ and $\;\sigma_{inter\text{-}veg}^{0}\;$ are simulated with the IEM-B model.

#### 2.3.2. Integral Equation Model Modified by Baghdadi (IEM-B)

Several models have been employed to relate soil properties to the backscattering process, such as the physics-based integral equation model proposed by [79]. The IEM simulates electromagnetic wave scattering from a randomly rough surface using radar sensor characteristics as inputs (frequency, polarization, and incidence angle) and a height autocorrelation function (exponential or Gaussian). Despite the satisfactory accuracy of the IEM in SAR signal behavior simulation across bare soil regions over other empirical and semi-empirical models, the roughness description parameter Lc exhibits some measurement incertitude.

Therefore, references [80,81,82,83] proposed an updated version of IEM, namely IEM-B, by replacing the Lc parameter with the fitting parameter Lopt as a function of the Hrms value and radar parameters (polarization and incidence angle θ). In this paper, we adopted the IEM-B model to generate bare soil backscattering values employing calibrated correlation lengths in the C-band in VV polarization (C-VV) and the L-band in HH polarization (L-HH), as expressed in Equations (9) and (10), respectively:

(9) $$\mathrm{Lopt}\left(\mathrm{Hrms},\;\theta ,\mathrm{CVV}\right)=1.281+0.134\times {\left(\sin(0.19\times \theta )\right)}^{-1.59}\times \mathrm{Hrms}$$

(10) $$\mathrm{Lopt}\left(\mathrm{Hrms},\;\theta ,\;\mathrm{LHH}\right)=2.6590\times \theta^{-1.4493}+3.0484\;\mathrm{Hrms}\times \theta^{-0.8044}$$

where θ  is the incidence angle in degrees in Equation (9) and θ  is the incidence angle in radian in Equation (10) and Lopt and Hrms are expressed in cm. The formulation of Lopt was obtained with the Gaussian correlation function. 

#### 2.3.3. Statistical Precision Parameters

Linear correlation coefficient R was adopted to evaluate the strength of the linear relationships between the radar signals and in situ measurements, as expressed in Equation (11). To assess the accuracy of model calibration and validation, we considered the root mean square error (RMSE) and bias, as expressed in Equations (12) and (13), respectively:

(11) $$R=\sqrt{\frac{\left(\sum_{i}^{N}\left(x_{i}-\bar{x}\right)\left(y_{i}-\bar{y}\right)\right)²}{\sum_{i}^{N}\left(x_{i}-\bar{x}\right)²\;\sum_{i}^{N}\left(y_{i}-\bar{y}\right)²}}$$

(12) $$\mathrm{Bias}=\frac{1}{N\;}\sum_{i=0}^{N}\left(x_{i}-y_{i}\right)$$

(13) $$\mathrm{RMSE}=\sqrt{\frac{1}{N}\;\sum_{i=0}^{N}{\left(x_{i}-y_{i}\right)}^{2}}$$

where $x_{i}$ and $y_{i}$ are the values of individual samples collected at points indexed with sample number i in Equation (11). $x_{i}\;$ and $y_{i}$ are the predicted and measured values, respectively, of sample i among *N* total data samples in Equations (12) and (13).

## 3. Results

### 3.1. Radar (Advanced Land Observing Satellite-2 (ALOS-2) and Sentinel-1) Sensitivity to Soil Moisture 

**Figure 7:**
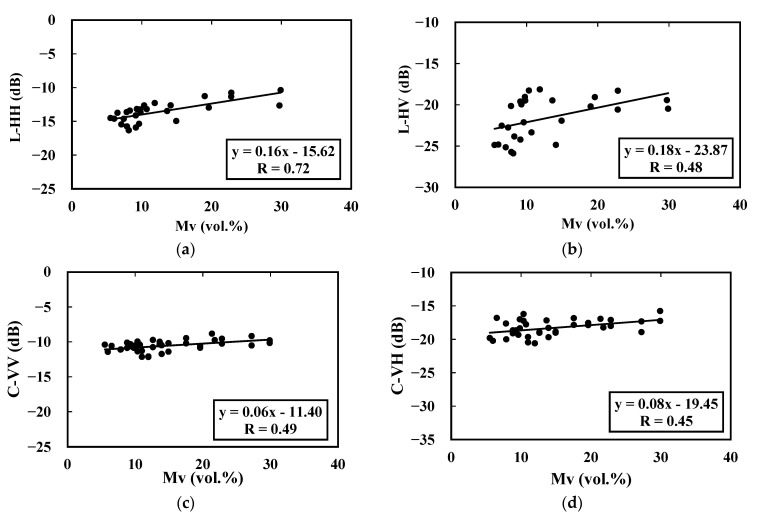
ALOS-2 and Sentinel-1 data as a function of soil moisture: L-band in (**a**) HH and (**b**) HV polarizations and C-band in dual-polarizations (**c**) VV and (**d**) VH.

In this section, we evaluate the influence of soil moisture on the L- and C-band radar signals at the different pepper growth stages, where the pepper height value varies between 0.17 and 0.64 m, the LAI value fluctuates between 0.07 and 1.75 and Fc is less than 0.5. The objective is to conduct a preliminary analysis to detect possible soil moisture effects in high-heterogeneity context of soil moisture and vegetation cover. Figure 7 shows the linear relationships between the ALOS-2 and Sentinel-1 data and soil moisture values calculated as mentioned in the second section based on Equation (2). Regarding the C-band data, we observe a limited correlation between radar data and soil moisture. The correlation coefficient values throughout the pepper growth cycle are lower than 0.49, revealing the limited sensitivity of the C-band to soil moisture, with a slope value varying between 0.06 dB/vol.% and 0.08 dB/vol.% in VV and VH polarizations, respectively. 

The initial results confirm the sensitivity of the L-band data to the average soil moisture. The sensitivity of the radar signals to soil moisture is approximately identical between the HH and HV polarizations, at 0.16 dB/vol.% and 0.18 dB/vol.%, respectively. However, the correlation is much stronger in the case of HH data. This can be explained by the volume scattering effect of the vegetation cover, which is much greater in the case of cross-polarization.

### 3.2. Radar (ALOS-2 and Sentinel-1) Sensitivity to Vegetation 

**Figure 8:**
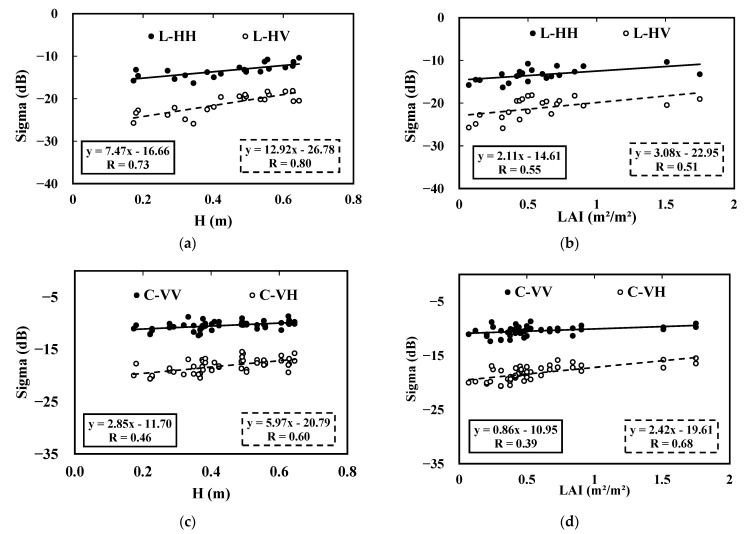
SAR signals as a function of biophysical vegetation parameters: L-band data as function of (**a**) H and (**b**) LAI and C-band data as function of (**c**) H and (**d**) LAI.

Figure 8 presents the linear relationships between the SAR signals and pepper biophysical parameters, i.e., vegetation height, and LAI. A high correlation characterizes the L-band data as a function of the vegetation height. The correlation coefficients range from 0.80 to 0.73 in the L-HV and L-HH configurations, respectively, with lower correlation coefficient values for the Sentinel-1 data varying between 0.6 and 0.46 in the C-VH and C-VV configurations, respectively. 

Regarding the LAI parameter, radar sensitivity increases from 2.11 dB.m²/m² to 3.08 dB.m²/m² using L-HH and L-HV, respectively. C-band sensitivity increases from 0.86 dB.m²/m² in VV polarization to 2.42 dB.m²/m² in VH polarization. We also observe some sensitivity in the L-HH, L-HV, C-VV and C-VH configurations but with low R values of 0.55, 0.51, 0.39 and 0.68, respectively. This could be explained by the fact that LAI information is mainly related to the upper canopy layer and corresponds to a mean value comprehensively without reflecting the heterogenous spatial vegetation cover. 

The vegetation height information reflects the canopy structure the most. Therefore, the L-band exhibits a higher sensitivity to this parameter (7.47 dB/m and 12.92 dB/m for HH and HV polarizations, respectively) thanks to its greater wave penetration. 

The C-band sensitivities to vegetation height vary between 2.85 dB/m and 5.97 dB/m for VV and VH polarizations, respectively. Within all contexts, a higher sensitivity to vegetation of the cross-polarization signals in the L-band or C-band is observed. This is related to their notable response to vegetation volume scattering. The abovementioned results emphasize the higher sensitivity of the SAR data in the cross-polarization configuration than that in the co-polarization configuration (L-HH and C-VV), as mentioned in [84].

The comparison between the L- and C-band behaviors reveals that the radar signals increase with increasing pepper development, as manifested by the increase of radar signal as function of H and LAI. Macelloni et al. [85] exanimated low-frequency radar behavior in covered fields as a function of the crop leaf nature, where L- and C-band SAR data increased as a function of the LAI for broad-leaf crops and decreased for narrow-leaf crops.

The same behavior was observed in previous studies using C-band [44,49,86] and X-band SAR data [63]. According to the results obtained, we investigated and analyzed the potential of co-polarized SAR data, namely, HH polarization data in the L-band and VV polarization data in the C-band, to limit the volume scattering effect on soil moisture retrieval in the following sections.

### 3.3. Calibration and Validation of the Modified WCM

In this section, we simulate the radar signal behavior in pepper fields through calibration and validation of the proposed model, as expressed in Equations (4) and (5). Based on the radar sensitivity results in Section 3.2, we adopt the vegetation height as a pepper descriptor in the following sections.

Modified WCM calibration and validation were carried out through the threefold cross-validation method due to the limited data samples (30 samples for L-band data and 32 samples for C-band data). This method consisted of a resampling procedure starting with random shuffling of the dataset followed by splitting into three partitions of equal size. With each partition, we calibrated the proposed model on 2/3 of the whole dataset, and we validated the model against 1/3 of the dataset. In each step, we evaluated the method performance by calculating the statistical parameters of the bias and RMSE.

Hence, A and B were calibrated three times. We only retained the most accurate calibration results in the subsequent steps. The A parameter values varied between 0.27 and 0.5 and B values fluctuated between 0.5 and 2.68, for L-HH and C-VV, respectively. The best WCM calibration performance was characterized by RMSE values ranging from 0.58 dB to 1.33 dB for the C-VV and L-HH data, respectively. The slightly higher RMSE in the L band could be explained by a greater roughness effect and surely a greater sensitivity to the entire canopy structure at this frequency band. 

**Figure 9:**
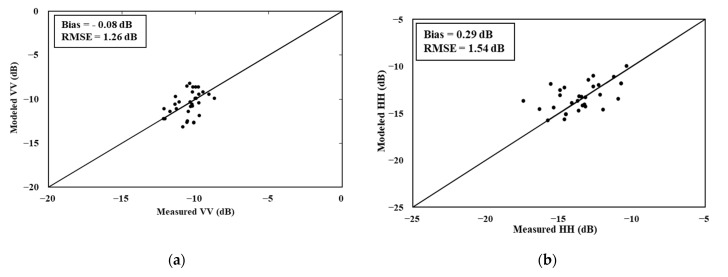
Comparison of the radar data and simulated backscattering coefficients in the pepper fields: (**a**) C-VV configuration and (**b**) L-HH configuration.

Figure 9 shows the validation results with an intercomparison of the SAR signals and backscattering coefficient model simulations. The RMSE value ranges from 1.26 dB to 1.54 dB, and the bias value fluctuates between −0.08 dB and 0.29 dB for the C-VV and L-HH data, respectively.

### 3.4. Radar Backscattering Simulations with the Modified WCM

In this section, we analyze the behavior of the backscattering coefficient as function of soil moisture using the calibrated modified WCM in the case of pepper fields, considering a large range of vegetation and soil conditions: bare soil moisture ranging from 5 to 40 vol.%, underlying soil moisture ranging from 10 to 40 vol.%, pepper height ranging from 0.1 to 0.7 m, and vegetation cover fraction ranging from 0.1 to 0.6. Table 3 summarizes all these parameters values. In this case, the soil moisture variation is principally controlled by irrigation and rainfall events. For instance, the increase in bare soil moisture is induced by rainfall, which generally increases the field average soil moisture content in the bare soil and pepper-covered soil parts. Furthermore, irrigation events increase the vegetation-influenced soil moisture. Therefore, we assume that the bare soil moisture level is always equal to or lower than the pepper plant-influenced soil moisture level to ensure the representativeness of the proposed model simulations.

In all simulations, we consider the hypothesis of constant roughness parameters (Hrms = 0.8 cm and Lc = 5 cm) as input into the IEM-B model to minimize the roughness effect. The principle of this analysis encompasses fixing a given condition and varying the other conditions to evaluate the slope of the linear relationships between soil moisture and modeled backscattering coefficients in the L-HH and C-VV configurations. We simulate the SAR backscattering coefficients with constant values of the cover fraction. At each value of the pepper coverage fraction, we varied the vegetation height level and recorded the slope (dB/vol.%). 

**Figure 10:**
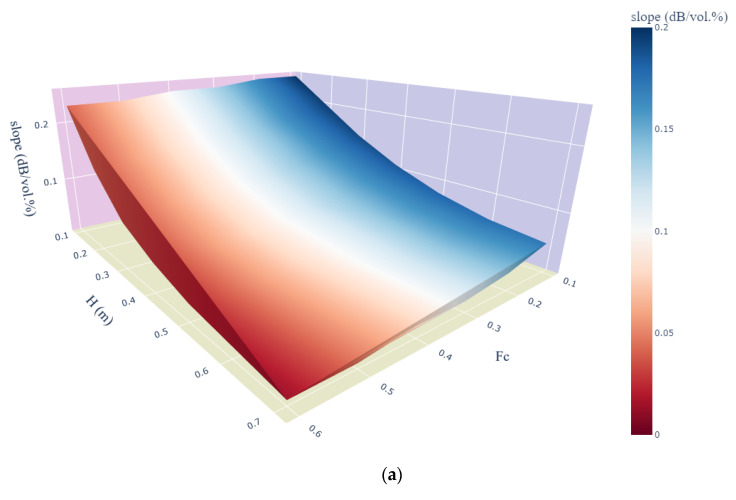

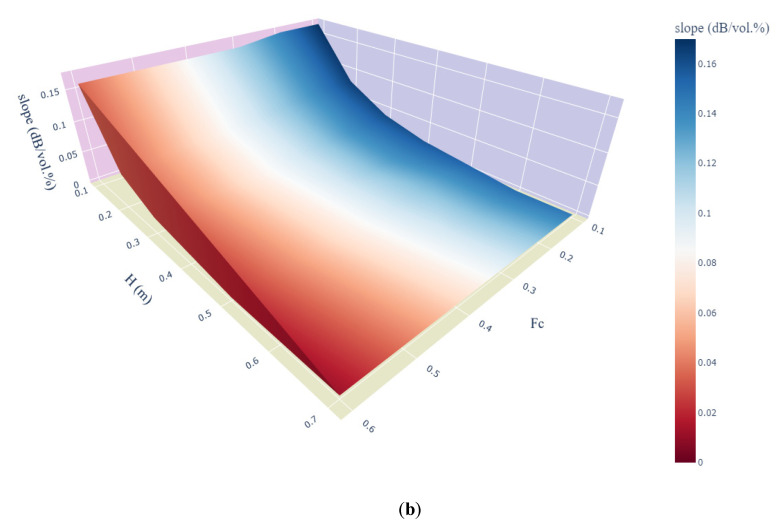
Three-dimensional surface scatter plot of the modified WCM sensitivities to soil moisture as a function of the pepper vegetation height and cover fraction using (**a**) L-HH and (**b**) C-VV data.

To better investigate the model sensitivity with vegetation parameter variation, we generated 3D surface scatter plots of the modified WCM sensitivity to the different soil moisture values according to the above established linear relationships as a function of the cover fraction and vegetation height (Figure 10) under different soil moisture conditions. With the use of L-HH data (Figure 10a), a high sensitivity was found at a low vegetation cover fraction and small height despite the smaller slope values starting at a height of 0.5 m and an Fc value of 0.4. Regarding C-VV data (Figure 10b), the model sensitivity decreased from 0.2 dB/vol.% at low *Fc* and H values to 0 dB/vol.% at an *Fc* value of 0.3 and a pepper height of 0.3 m. 

**Figure 11:**
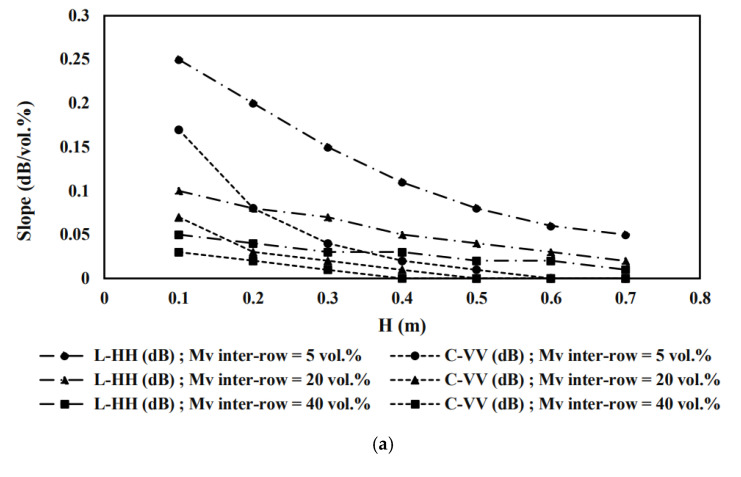

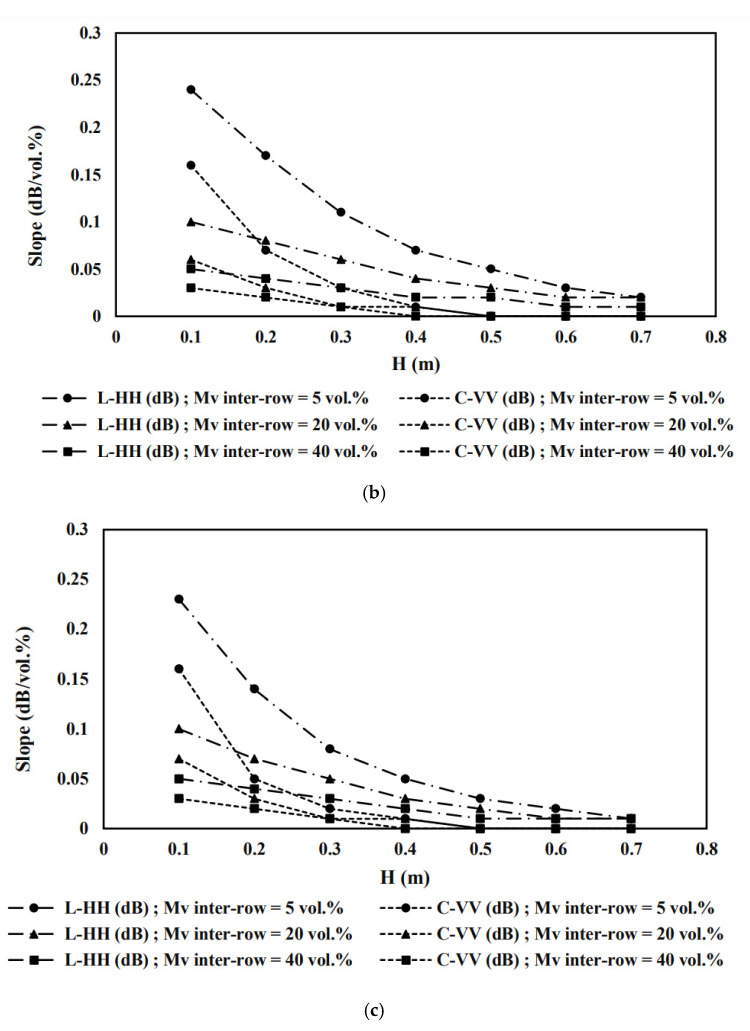
Scatterplots of the modified WCM sensitivities to soil moisture as a function of the pepper vegetation height (H) using L-HH and C-VV data in three different contexts of inter-rows soil moisture (5 vol.%, 20 vol.% and 40 vol.%) with constant values of cover fraction (**a**) *Fc* = 0.1, (**b**) *Fc* = 0.3 and (**c**) *Fc* = 0.6.

To further investigate the evolution of model sensitivities as function of vegetation height and cover fraction, we have chosen to present 2D profiles extracted from Figure 10 where Fc values are equal to 0.1, 0.3 and 0.6. At each constant value of Fc, we scatterplot, the proposed model sensitivity to soil moisture as function of pepper height using the SAR data (L-HH and C-VV) under inter-row soil moisture values equal to 5 vol.%, 20 vol.% and 40 vol.% (Figure 11). 

Figure 11a represents simulation results when cover fraction is equal to 0.1 in different soil moisture contexts. Globally, the model sensitivities decrease as function of pepper height increase. Using L-HH data, the model sensitivities vary from 0.25 to 0.10 dB/vol.%. 

Using C-VV data, the simulation slope fluctuates between 0.17 dB/vol.% and 0 dB/vol.%. For a vegetation height equal to 0.1 m, L-HH sensitivities vary from 0.25 to 0.05 dB/vol.% regarding a slope between 0.17 and 0.03 dB/vol.% using C-VV for $Mv_{inter\text{-}row}\;$ values equal to 5 and 40 vol.%, respectively. When the vegetation height increases to 0.4 m, L-HH sensitivity still higher than 0.1 dB/vol.% for inter-row soil moisture equal to 5 vol.%, regarding slopes lower than of 0.03 dB/vol.% for C-VV case under the different $Mv_{inter\text{-}row}$ contexts. 

Pepper height reaches its maximum at 0.7 m. For this case, L-HH signal slopes vary from 0.05 dB/vol.% to 0.01 dB/vol.% regarding zero values for C-VV simulations. With the increase of pepper’s height, the dynamic of model sensitivities, using L-HH data, decreases from 0.20 dB/vol.% to 0.04 dB/vol.% for H values equal to 0.1 m and 0.7 m, respectively. We notice low sensibility characterizing C-VV simulations where sensitivity decrease from 0.14 dB/vol.% to be null, for vegetation heights equal to 0.1 m and 0.7 m, respectively. Under low vegetation cover fraction (*Fc* = 0.1), the comparison between L-HH and C-VV simulation results reveals that the modified WCM, using L-HH data, remains sensitive to soil moisture as function of the increase pepper height and the inter-rows soil water content. However, the proposed model using C-VV becomes insensitive to soil moisture with the vertical development of pepper plants. This C-band behavior is induced from a strong attenuation of the signal over peppers. 

According to the Figure 11b, the same trends were noticed for L-HH and sensitivities as shown in the Figure 11a but with a lower sensitivity dynamic for a Fc value equal to 0.3. Using L-HH data, the slope variation is between 0.19 dB/vol.%, 0.05 dB/vol.% and 0.01 dB/vol.% when H values are equal to 0.1 m, 0.4 m, and 0.7 m, respectively, under three contexts of the inter-row soil moisture. Using C-VV data, sensitivity dynamic starts from 0.13 dB/vol.% to be 0.01 dB/vol. % and then null for three H values equal to 0.1 m, 0.4 m, and 0.7 m, respectively. Figure 11c shows the scatterplot of the model sensitivities as function of pepper height where cover fraction is equal to 0.6 under the different context of inter-row soil moisture. The sensitivity to soil moisture fluctuates between 0.23 dB/vol.% and 0.01 dB/vol.% for L-HH data and between 0.16 dB/vol.% and 0.03 dB/vol.% for C-VV data, for a vegetation height value equal to 0.1 m and $Mv_{inter\text{-}row}$ values varying between 5 and 40 vol.%. With the increase of pepper height, sensitivity variation, using C-VV, decreases to be null where H values reach 0.4 m. 

The examination of the three profiles reveals the following conclusions. The modified WCM sensitivity decreases as function of the increase of pepper biophysical parameters: vertically (pepper height) and horizontally (cover fraction) and inter-row soil moisture values. For constant values of cover fraction values, the dynamics of the sensitivity to soil moisture decreases as function of the increase of pepper height. This decrease may indicate the domination of bare soil the total backscattering mechanism until the pepper height reaches the value of 0.3 m for the C-VV signal and 0.5 m for the L-HH signal. Subsequently, vegetation backscattering contribution increases with the pepper growth regarding the decrease of bare soil impact. 

The comparison of simulation results underlines that model sensitivity based on C-VV data decreases more rapidly than does that based on L-HH data as a function of the pepper cover fraction and height and the soil water content. The L-HH data sensitivity declines and remains relatively higher than the C-VV data sensitivity given the same values of Fc and H. The aforementioned results also highlight the potential of the modified WCM to simulate SAR signals in the pepper reference fields. Conversely, the proposed model potential should be further explored considering other heterogeneous plants under drip irrigation via a comparison to the present study.

## 4. Conclusions

This study proposes an analysis of the potential of SAR data for soil moisture retrieval in drip-irrigated pepper fields in semiarid areas at the center of Tunisia, i.e., the Kairouan Plain, using L- and C-band SAR data. By considering that 15% of the field area is influenced by drip irrigation and that the remaining area is dominated by non-irrigated bare soil, we analyzed the sensitivity of radar signal to the weighted soil moisture content (Mv). According to the initial sensitivity analysis to detect possible soil moisture effects under a high soil moisture heterogeneity where the pepper height varied between 0.17 and 0.64 m, the LAI value fluctuated between 0.07 and 1.75 and Fc was lower than 0.5, the results revealed that the L-HH data were more sensitive than were the C-VV data to the soil water content. The sensitivity decreased from 0.16 dB/vol.% in L-HH case to 0.06 dB/vol.% using C-VV. Regarding cross-polarized data, volume scattering generated noise, which impacted the sensitivity of SAR signal where R values were lower than 0.48 for both the L-HV and C-VH data. The radar signal sensitivity was investigated as a function of two pepper biophysical properties: vegetation height and LAI. 

Radar signal sensitivity analysis of these pepper biophysical parameters revealed a high correlation of the linear relationships between the SAR data and vegetation height compared to the LAI parameter. Based on the results, we proposed a modified version of the WCM. In this model, we considered the vegetation height as a vegetation descriptor and we used the cover fraction to separate the backscattering mechanisms of the bare soil and vegetation row parts. To simulate SAR signals, the suggested model parameters required calibration and validation via the three-fold cross-validation method. The best calibration RMSE values ranged from 0.58 dB for the C-VV data to 1.33 dB for the L-HH data. The validation accuracy parameters, namely, the bias and RMSE, varied between 0.29 dB and 1.54 dB for the L-HH data and between −0.08 dB and 1.26 dB for the C-VV data.

We evaluated the sensitivity of the suggested model to soil moisture. The results highlighted the potential of the modified version of the WCM to simulate ALOS-2 and Sentinel-1 signals over inter-pixel heterogenous soil moisture context. This potential is impacted by the inter vegetation rows moisture content and the variation of vegetation biophysical parameters (pepper height and vegetation cover fraction). Using L-HH data, the proposed model remained sensitive to soil moisture where sensitivity values vary between 0.25 dB/vol.% and 0.08 dB/vol.% at a vegetation height and a cover fraction values less than 0.5 m and 0.4, respectively, where inter-row soil moisture value is equal to 5 vol.%. Under the same soil moisture context, the model sensitivity, using C-VV data, decreased from 0.17 dB/vol.% to 0.08 dB/vol.% at a vegetation height less than 0.3 m and a cover fraction value lower than 0.3. The aforementioned vegetation biophysical parameters thresholds vary as function of the inter-row vegetation soil moisture contexts. 

One of the limitations of the proposed semi-empirical approach is its dependence on the database used and the study objectives. Hence, the suggested approach should be tested at other sites. The inversion process of the modified WCM coupled with the IEM-B model for soil moisture retrieval purposes is complex to realize. Therefore, machine-learning algorithms, such as artificial neural networks (ANNs), will be applied in future work to estimate the soil water content.

## Figures and Tables

**Table 1:** Available synthetic aperture radar (SAR) image characteristics acquired in 2020 by Advanced Land Observing Satellite-2 (ALOS-2) in Horizontal-Horizontal (HH) and Horizontal-Vertical (HV) polarizations and Sentinel-1 A (S-1 A) and B (S-1 B) in Vertical-Vertical (VV) and Vertical- Horizontal (VH) polarizations.

Date	Incidence Angle	Sensor	Polarization Scheme	Ascending/ Descending State
8 June 2020	32.5°	ALOS-2	HH + HV	Descending
17 June 2020	39°	S-1 A	VV + VH	Ascending
22 June 2020	32.5°	ALOS-2	HH + HV	Descending
24 June 2020	39°	S-1 B	VV + VH	Descending
5 July 2020	39°	S-1 B	VV + VH	Ascending
6 July 2020	39°	S-1 B	VV + VH	Descending
20 July 2020	32.5°	ALOS-2	HH + HV	Descending
23 July 2020	39°	S-1 A	VV + VH	Ascending
3August 2020	32.5°	ALOS-2	HH + HV	Descending
4 August 2020	39°	S-1 A	VV + VH	Ascending
16 August 2020	39°	S-1 A	VV + VH	Ascending
17 August 2020	32.5°	ALOS-2	HH + HV	Descending
17 August 2020	39°	S-1 A	VV + VH	Descending

**Table 2:** The minimum and maximum values of in situ measurements during the data gathering campaign in 2020. Root mean square surface height Hrms, correlation length Lc, soil moisture Mv (inter-row soil moisture $Mv_{inter\text{-}row}$ and vegetation-influenced moisture $Mv_{veg\text{-}row}$) and pepper parameters (height, Leaf Area Index (LAI), and cover fraction (*Fc*)).

**Date**	Measurements						
	Hrms (cm)	Lc (cm)	$Mv_{inter\text{-}row}\;(\mathrm{vol}.\%)$	$Mv_{veg\text{-}row}\;(\mathrm{vol}.\%)$	Height (m)	LAI (m²/m²)	*Fc*
8 June 2020	[1.84–2.54]	[2.98–7.40]	[4.90–6.10]	[12.50–17.50]	[0.19–0.40]	[0.15–0.18]	[0.20–0.33]
17 June 2020	[1.84–2.54]	[2.98–7.40]	[5.70–10.70]	[9.40–23.50]	-	-	-
22 June 2020	-	-	[5.50–21.10]	[5.60–27.60]	[0.17–0.41]	[0.07–0.56]	[0.08–0.43]
24 June 2020	-	-	[5.50–21.10]	[5.60–27.60]	[0.17–0.41]	[0.07–0.56]	[0.08–0.43]
5 July 2020	[1.50–2.54]	[2,98–7.40]	[5.80–27]	[6.80–28.50]	[0.22–0.42]	[0.20–0.45]	[0.20–0.50]
6 July 2020	-	-	[5.80–27]	[6.80–28.50]	[0.22–0.42]	[0.20–0.45]	[0.20–0.50]
20 July 2020	[1.50–2.54]	[2.98–7.40]	[6.40–31.40]	[8.90–30.90]	[0.27–0.55]	[0.30–0.71]	[0.21–0.38]
23 July 2020	[1.50–2.54]	[2.98–7.40]	-	-	[0.22–0.56]	[0.32–0.71]	[0.25–0.38]
3August 2020	[1.50–2.54]	[2.98–7.40]	[6.80–24.10]	[9.80–31]	[0.35–0.63]	[0.38–0.90]	[0.33–0.46]
4 August 2020	-	-	[6.80–20.10]	[9.80–31]	-	-	-
16 August 2020	[1.50–2.54]	[2.98–7.40]	[5.30–29.50]	[10.80–32.10]	[0.39–0.64]	[0.50–1.75]	[0.17–0.46]
17 August 2020	-	-	[5.30–29.50]	[10.80–32.10]	[0.39–0.64]	[0.50–1.75]	[0.17–0.46]

**Table 3:** The parameter values variation used in model simulation: soil moisture (vol.%) and pepper parameters (height and cover fraction *Fc*).

Parameter		Values
Soil moisture	$Mv_{inter\text{-}row}$ (vol.%)	5, 10, 20, 30, 40
	$Mv_{veg\text{-}row}$ (vol.%)	10, 20, 30, 40
Biophysical parameters	Height (m)	0.1, 0.2, 0.3, 0.4, 0.5, 0.6, 0.7
	*Fc*	0.1, 0.2, 0.3, 0.4, 0.5, 0.6

## Data Availability

Data are available in Theia Land site https://www.theia-land.fr/ (accessed on 8 January 2022).

## References

[1] Cai X., Rosegrant M.W. (2002). Global Water Demand and Supply Projections: Part 1. A Modeling Approach. Water Int..

[2] FAO, FIDA, OMS, PAM et UNICEF (2021). L’État de la Sécurité Alimentaire et de la Nutrition dans le Monde 2021. Transformer les Systèmes Alimentaires Pour que la Sécurité Alimentaire, une Meilleure Nutrition et une Alimentation Saine et Abordable Soient une Réalité Pour Tous.

[3] Abioye E.A., Abidin M.S.Z., Mahmud M.S.A., Buyamin S., AbdRahman M.K.I., Otuoze A.O., Ramli M.S.A., Ijike O.D. (2020). IoT-based monitoring and data-driven modelling of drip irrigation system for mustard leaf cultivation experiment. Inf. Process. Agric..

[4] Tramblay Y., Koutroulis A., Samaniego L., Vicente-Serrano S.M., Volaire F., Boone A., Le Page M., Llasat M.C., Albergel C., Burak S. (2020). Challenges for drought assessment in the Mediterranean region under future climate scenarios. Earth-Sci. Rev..

[5] Pereira L.S., Oweis T., Zairi A. (2002). Irrigation management under water scarcity. Agric. Water Manag..

[6] Rosa L., Chiarelli D.D., Rulli M.C., Angelo J.D., Odorico P.D. (2020). Global agricultural economic water scarcity. Sci. Adv..

[7] Amiri Z., Gheysari M., Mosaddeghi M.R., Amiri S., Tabatabaei M.S., Ozdogan M., Yang Y., Allez G., Cervantes C., Wolff P. (2010). Remote sensing of irrigated agriculture: Opportunities and challenges. Remote Sens..

[8] Massari C., Modanesi S., Dari J., Gruber A., De Lannoy G.J.M., Girotto M., Quintana-Seguí P., Le Page M., Jarlan L., Zribi M. (2021). A review of irrigation information retrievals from space and their utility for users. Remote Sens..

[9] Motte E., Zribi M., Fanise P., Egido A., Darrozes J., Al-Yaari A., Baghdadi N., Baup F., Dayau S., Fieuzal R. (2016). GLORI: A GNSS-R Dual Polarization Airborne Instrument for Land Surface Monitoring. Sensors.

[10] El Hajj M., Baghdadi N., Zribi M., Bazzi H. (2017). Synergic use of Sentinel-1 and Sentinel-2 images for operational soil moisture mapping at high spatial resolution over agricultural areas. Remote Sens..

[11] Zribi M., André C., Decharme B. (2008). A method for soil moisture estimation in Western Africa based on the ERS scatterometer. IEEE Trans. Geosci. Remote Sens..

[12] Sekertekin A., Marangoz A.M., Abdikan S., Esetlili M.T. Preliminary results of estimating soil moisture over bare soil using full-polarimetric ALOS-2 data. Proceedings of the International Archives of the Photogrammetry, Remote Sensing and Spatial Information Sciences—ISPRS Archives.

[13] Wang C., Qi J., Moran S., Marsett R. (2004). Soil moisture estimation in a semiarid rangeland using ERS-2 and TM imagery. Remote Sens. Environ..

[14] Gherboudj I., Magagi R., Berg A.A., Toth B. (2011). Soil moisture retrieval over agricultural fields from multi-polarized and multi-angular RADARSAT-2 SAR data. Remote Sens. Environ..

[15] Attarzadeh R., Amini J., Notarnicola C., Greifeneder F. (2018). Synergetic use of Sentinel-1 and Sentinel-2 data for soil moisture mapping at plot scale. Remote Sens..

[16] Bao Y., Lin L., Wu S., Abdalla K., Deng K., Petropoulos G.P. (2018). Surface soil moisture retrievals over partially vegetated areas from the synergy of Sentinel-1 and Landsat 8 data using a modi fi ed water-cloud model. Int. J. Appl. Earth Obs. Geoinf..

[17] Hosseini M., McNairn H., Mitchell S., Dingle Robertson L., Davidson A., Homayouni S. (2019). Synthetic aperture radar and optical satellite data for estimating the biomass of corn. Int. J. Appl. Earth Obs. Geoinf..

[18] Zribi M., Muddu S., Bousbih S., Al Bitar A., Tomer S.K., Baghdadi N., Bandyopadhyay S. (2019). Analysis of L-band SAR data for soil moisture estimations over agricultural areas in the tropics. Remote Sens..

[19] Bazzi H., Baghdadi N., Fayad I., Zribi M., Belhouchette H., Demarez V. (2020). Near real-time irrigation detection at plot scale using sentinel-1 data. Remote Sens..

[20] Ezzahar J., Ouaadi N., Zribi M., Elfarkh J., Aouade G., Khabba S., Er-Raki S., Chehbouni A., Jarlan L. (2020). Evaluation of backscattering models and support vector machine for the retrieval of bare soil moisture from sentinel-1 data. Remote Sens..

[21] Zhang L., Lv X., Chen Q., Sun G., Yao J. (2020). Estimation of surface soil moisture during corn growth stage from SAR and optical data using a combined scattering model. Remote Sens..

[22] Zribi M., Foucras M., Baghdadi N., Demarty J., Muddu S. (2021). A New Reflectivity Index for the Retrieval of Surface Soil Moisture from Radar Data. IEEE J. Sel. Top. Appl. Earth Obs. Remote Sens..

[23] Baghdadi N., Cresson R., El Hajj M., Ludwig R., La Jeunesse I. (2012). Estimation of soil parameters over bare agriculture areas from C-band polarimetric SAR data using neural networks. Hydrol. Earth Syst. Sci..

[24] Baghdadi N., Cresson R., Pottier E., Aubert M., Zribi M., Jacome A., Benabdallah S. (2012). A potential use for the C-band polarimetric SAR parameters to characterize the soil surface over bare agriculture fields. IEEE Trans. Geosci. Remote Sens..

[25] Aubert M., Baghdadi N.N., Zribi M., Ose K., El Hajj M., Vaudour E., Gonzalez-Sosa E. (2013). Toward an operational bare soil moisture mapping using terrasar-x data acquired over agricultural areas. IEEE J. Sel. Top. Appl. Earth Obs. Remote Sens..

[26] Baghdadi N.N., El Hajj M., Zribi M., Fayad I. (2016). Coupling SAR C-Band and Optical Data for Soil Moisture and Leaf Area Index Retrieval over Irrigated Grasslands. IEEE J. Sel. Top. Appl. Earth Obs. Remote Sens..

[27] El Hajj M., Baghdadi N., Cheviron B., Belaud G., Zribi M. (2016). Integration of remote sensing derived parameters in crop models: Application to the PILOTE model for hay production. Agric. Water Manag..

[28] Bai X., He B., Li X., Zeng J., Wang X., Wang Z., Zeng Y., Su Z. (2017). First assessment of Sentinel-1A data for surface soil moisture estimations using a coupled water cloud model and advanced integral equation model over the Tibetan Plateau. Remote Sens..

[29] Gao Q., Zribi M., Escorihuela M.J., Baghdadi N. (2017). Synergetic use of sentinel-1 and sentinel-2 data for soil moisture mapping at 100 m resolution. Sensors.

[30] Zribi M., Ciarletti V., Taconet O. (2000). Validation of a Rough Surface Model Based on Fractional Brownian Geometry with SIRC and ERASME Radar Data over Orgeval. Remote Sens. Environ..

[31] Whitt M.W., Ulaby F.T. (1994). Radar Response of Periodic Vegetation Canopies. Int. J. Remote Sens..

[32] Steele-Dunne S.C., McNairn H., Monsivais-Huertero A., Judge J., Liu P.W., Papathanassiou K. (2017). Radar Remote Sensing of Agricultural Canopies: A Review. IEEE J. Sel. Top. Appl. Earth Obs. Remote Sens..

[33] Karam M.A., Fung A.K., Antar Y.M.M. (1988). Electromagnetic Wave Scattering from Some Vegetation Samples. IEEE Trans. Geosci. Remote Sens..

[34] Karam M.A., Amar F., Fung A.K., Mougin E., Lopes A., Le Vine D.M., Beaudoin A. (1995). A microwave polarimetric scattering model for forest canopies based on vector radiative transfer theory. Remote Sens. Environ..

[35] Ulaby F.T., Sarabandi K., Mcdonald K., Whitt M., Dobson M.C. (1990). Michigan microwave canopy scattering model. Int. J. Remote Sens..

[36] De Roo R.D., Du Y., Ulaby F.T., Craig Dobson M. (2001). A semi-empirical backscattering model at L-band and C-band for a soybean canopy with soil moisture inversion. IEEE Trans. Geosci. Remote Sens..

[37] Du J., Shi J., Sun R. (2010). The development of HJ SAR soil moisture retrieval algorithm. Int. J. Remote Sens..

[38] Weiß T., Ramsauer T., Löw A., Marzahn P. (2020). Evaluation of Different Radiative Transfer Models for Microwave Backscatter Estimation of Wheat Fields. Remote Sens..

[39] Attema E.P.W., Ulaby F.T. (1978). Vegetation modeled as a water cloud. Radio Sci..

[40] Ulaby F.T., Allen C.T., Eger G., Kanemasu E. (1984). Relating the microwave backscattering coefficient to leaf area index. Remote Sens. Environ..

[41] Dabrowska-Zielinska K., Inoue Y., Kowalik W., Gruszczynska M. (2007). Inferring the effect of plant and soil variables on C- and L-band SAR backscatter over agricultural fields, based on model analysis. Adv. Space Res..

[42] Kumar K., Prasad K.S.H., Arora M.K. (2012). Estimation of water cloud model vegetation parameters using a genetic algorithm. Hydrol. Sci. J..

[43] Baghdadi N. (2017). Calibration of the Water Cloud Model at C-Band for Winter Crop Fields and Grasslands. Remote Sens..

[44] Bousbih S., Zribi M., Lili-Chabaane Z., Baghdadi N., El Hajj M., Gao Q., Mougenot B. (2017). Potential of sentinel-1 radar data for the assessment of soil and cereal cover parameters. Sensors.

[45] Li J., Wang S. (2018). Using SAR-derived vegetation descriptors in a water cloud model to improve soil moisture retrieval. Remote Sens..

[46] Ouaadi N., Jarlan L., Ezzahar J., Khabba S., Le Dantec V., Rafi Z., Zribi M., Frison P.L. Water Stress Detection over Irrigated Wheat Crops in Semi-Arid Areas Using the Diurnal Differences of Sentinel-1 Backscatter. Proceedings of the 2020 Mediterranean and Middle-East Geoscience and Remote Sensing Symposium (M2GARSS).

[47] He B., Xing M., Bai X. (2014). A synergistic methodology for soil moisture estimation in an alpine prairie using radar and optical satellite data. Remote Sens..

[48] Qiu J., Crow W.T., Wagner W., Zhao T. (2019). Effect of vegetation index choice on soil moisture retrievals via the synergistic use of synthetic aperture radar and optical remote sensing. Int. J. Appl. Earth Obs. Geoinf..

[49] Ayari E., Kassouk Z., Lili-Chabaane Z., Baghdadi N., Bousbih S., Zribi M. (2021). Cereal crops soil parameters retrieval using L-band ALOS-2 and C-band sentinel-1 sensors. Remote Sens..

[50] Paloscia S., Pettinato S., Santi E., Notarnicola C., Pasolli L., Reppucci A. (2013). Soil moisture mapping using Sentinel-1 images: Algorithm and preliminary validation. Remote Sens. Environ..

[51] Ouaadi N., Jarlan L., Ezzahar J., Zribi M., Khabba S., Bouras E., Frison P.L. Surface Soil Moisture Retrieval over Irrigated Wheat Crops in Semi-Arid Areas Using Sentinel-1 Data. Proceedings of the 2020 Mediterranean and Middle-East Geoscience and Remote Sensing Symposium (M2GARSS).

[52] Wang Z., Zhao T., Qiu J., Zhao X., Li R., Wang S. (2021). Microwave-based vegetation descriptors in the parameterization of water cloud model at L-band for soil moisture retrieval over croplands. GISci. Remote Sens..

[53] El Hajj M., Baghdadi N., Zribi M., Belaud G., Cheviron B., Courault D., Charron F. (2016). Soil moisture retrieval over irrigated grassland using X-band SAR data. Remote Sens. Environ..

[54] Graham A.J., Harris R. (2003). Extracting biophysical parameters from remotely sensed radar data: A review of the water cloud model. Prog. Phys. Geogr..

[55] Gao S., Niu Z., Huang N., Hou X. (2013). Estimating the Leaf Area Index, height and biomass of maize using HJ-1 and RADARSAT-2. Int. J. Appl. Earth Obs. Geoinf..

[56] Veloso A., Mermoz S., Bouvet A., Le Toan T., Planells M., Dejoux J.F., Ceschia E. (2017). Understanding the temporal behavior of crops using Sentinel-1 and Sentinel-2-like data for agricultural applications. Remote Sens. Environ..

[57] Vreugdenhil M., Wagner W., Bauer-Marschallinger B., Pfeil I., Teubner I., Rüdiger C., Strauss P. (2018). Sensitivity of Sentinel-1 backscatter to vegetation dynamics: An Austrian case study. Remote Sens..

[58] Gorrab A., Ameline M., Albergel C., Baup F. (2021). Use of sentinel-1 multi-configuration and multi-temporal series for monitoring parameters of winter wheat. Remote Sens..

[59] Bousbih S., Zribi M., El Hajj M., Baghdadi N. (2018). Soil Moisture and Irrigation Mapping in A Semi-Arid Region, Based on the Synergetic Use of Sentinel-1. Remote Sens..

[60] Tomer S.K., Al Bitar A., Sekhar M., Zribi M., Bandyopadhyay S., Sreelash K., Sharma A.K., Corgne S., Kerr Y. (2015). Retrieval and multi-scale validation of Soil Moisture from multi-temporal SAR Data in a semi-arid tropical region. Remote Sens..

[61] Zhu L., Walker J.P., Ye N., Rüdiger C. (2019). Roughness and vegetation change detection: A pre-processing for soil moisture retrieval from multi-temporal SAR imagery. Remote Sens. Environ..

[62] Pierdicca N., Pulvirenti L., Pace G. (2014). A prototype software package to retrieve soil moisture from sentinel-1 data by using a bayesian multitemporal algorithm. IEEE J. Sel. Top. Appl. Earth Obs. Remote Sens..

[63] El Hajj M., Baghdadi N., Belaud G., Zribi M., Cheviron B., Courault D., Hagolle O., Charron F. (2014). Irrigated grassland monitoring using a time series of TerraSAR-X and COSMO-SkyMed X-Band SAR data. Remote Sens..

[64] Fieuzal R., Marais Sicre C., Baup F. (2017). Estimation of corn yield using multi-temporal optical and radar satellite data and artificial neural networks. Int. J. Appl. Earth Obs. Geoinf..

[65] El Hajj M., Baghdadi N., Bazzi H., Zribi M. (2019). Penetration analysis of SAR signals in the C and L bands for wheat, maize, and grasslands. Remote Sens..

[66] Mirsoleimani H.R., Sahebi M.R., Baghdadi N., El Hajj M. (2019). Bare soil surface moisture retrieval from sentinel-1 SAR data based on the calibrated IEM and dubois models using neural networks. Sensors.

[67] Hamze M., Baghdadi N., El Hajj M.M., Zribi M., Bazzi H., Cheviron B., Faour G. (2021). Integration of L-Band Derived Soil Roughness into a Bare Soil Moisture Retrieval Approach from C-Band SAR Data. Remote Sens..

[68] Svoray T., Shoshany M. (2002). SAR-based estimation of areal aboveground biomass (AAB) of herbaceous vegetation in the semi-arid zone: A modification of the water-cloud model. Int. J. Remote Sens..

[69] Xing M., He B., Ni X., Wang J., An G., Shang J., Huang X. (2019). Retrieving Surface Soil Moisture over Wheat and Soybean Fields during Growing Season Using Modified Water Cloud Model from Radarsat-2 SAR Data. Remote Sens..

[70] Davidson M., Chini M., Dierking W., Djavidnia S., Haarpaintner J., Hajduch G., Laurin G.V., Lavalle M., López-Martinez C., Nagler T. (2019). Copernicus L-band SAR Mission Requirements Document.

[71] Amiri Z., Gheysari M., Mosaddeghi M.R., Amiri S., Tabatabaei M.S. (2021). An Attempt to Find a Suitable Place for Soil Moisture Sensor in a Drip Irrigation System. Inf. Process. Agric..

[72] Amri R. (2013). Estimation Régionale de L’évapotranspiration sur la Plaine de Kairouan (Tunisie) à Partir de Données Satellites Multi-capteurs École. Ph.D. Thesis.

[73] Leduc C., Ammar S.B.E.N., Favreau G., Beji R., Virrion R. (2007). Impacts of hydrological changes in the Mediterranean zone: Environmental modifications and rural development in the Merguellil catchment, central Tunisia. Hydrol. Sci. J./J. Des Sci. Hydrol..

[74] Zribi M., Chahbi A., Shabou M., Lili-Chabaane Z., Duchemin B., Baghdadi N., Amri R., Chehbouni A. (2011). Soil surface moisture estimation over a semi-arid region using ENVISAT ASAR radar data for soil evaporation evaluation. Hydrol. Earth Syst. Sci..

[75] Bousbih S., Zribi M., Pelletier C., Gorrab A., Lili-Chabaane Z., Baghdadi N., Aissa N.B., Mougenot B. (2019). Soil texture estimation using radar and optical data from Sentinel-1 and Sentinel-2. Remote Sens..

[76] Rouse J.W., Haas R.H., Schell J.A., Deering D.W. (1973). Monitoring the Vernal Advancement and Retrogradation (Green Wave Effect) of Natural Vegetation.

[77] Gutman G., Ignatov A. (1998). The derivation of the green vegetation fraction from NOAA/AVHRR data for use in numerical weather prediction models. Int. J. Remote Sens..

[78] Ulaby F.T. (1975). Radar Response to Vegetation. IEEE Trans. Antennas Propag..

[79] Fung A.K., Li Z., Chen K.S. (1992). Backscattering from a Randomly Rough Dielectric Surface. IEEE Trans. Geosci. Remote Sens..

[80] Baghdadi N., Gherboudj I., Zribi M., Sahebi M., King C., Bonn F. (2004). Semi-empirical calibration of the IEM backscattering model using radar images and moisture and roughness field measurements. Int. J. Remote Sens..

[81] Baghdadi N., Holah N., Zribi M. (2006). Soil moisture estimation using multi-incidence and multi-polarization ASAR data. Int. J. Remote Sens..

[82] Baghdadi N., Abou Chaaya J., Zribi M. (2011). Semiempirical calibration of the integral equation model for SAR data in C-Band and cross polarization using radar images and field measurements. IEEE Geosci. Remote Sens. Lett..

[83] Baghdadi N., Zribi M., Paloscia S., Verhoest N.E.C., Lievens H., Baup F., Mattia F. (2015). Semi-empirical calibration of the integral equation model for co-polarized L-band backscattering. Remote Sens..

[84] Inoue Y., Kurosu T., Maeno H., Uratsuka S., Kozu T., Dabrowska-Zielinska K., Qi J. (2002). Season-long daily measurements of multifrequency (Ka, Ku, X, C, and L) and full-polarization backscatter signatures over paddy rice field and their relationship with biological variables. Remote Sens. Environ..

[85] Macelloni G., Paloscia S., Pampaloni P., Marliani F., Gai M. (2001). The relationship between the backscattering coefficient and the biomass of narrow and broad leaf crops. IEEE Trans. Geosci. Remote Sens..

[86] Fieuzal R., Baup F., Marais-Sicre C. (2013). Monitoring Wheat and Rapeseed by Using Synchronous Optical and Radar Satellite Data—from Temporal Signatures to Crop Parameters Estimation. Adv. Remote Sens..

